# Potentially preventable visits to the emergency department in older adults: Results from a national survey in Italy

**DOI:** 10.1371/journal.pone.0189925

**Published:** 2017-12-21

**Authors:** Beatrice Gasperini, Antonio Cherubini, Francesca Pierri, Pamela Barbadoro, Massimiliano Fedecostante, Emilia Prospero

**Affiliations:** 1 Department of Biomedical Sciences and Public Health, Section of Hygiene, Preventive Medicine and Public Health, Università Politecnica delle Marche, Ancona, Italy; 2 Department of Geriatrics and Rehabilitation, Santa Croce Hospital, Azienda Ospedaliera Ospedali Riuniti Marche Nord, Fano, Italy; 3 Geriatria, Accettazione geriatrica e Centro di ricerca per l’invecchiamento, IRCCS-INRCA, Ancona, Italy; 4 Department of Economy, Section of Statistics, University of Perugia, Perugia, Italy; University of Brescia, ITALY

## Abstract

**Background:**

Despite older adults use emergency department more appropriately than other age groups, there is a significant share of admissions that can be considered potentially preventable.

**Objective:**

To identify socio-demographic characteristics and health care resources use of older adults admitted to emergency department for a potentially preventable visit.

**Design:**

Data come from the Multipurpose Survey “Health conditions and use of health services”, edition 2012–2013. A stratified multi-stage probability design was used to select a sample using municipal lists of households.

**Subject:**

50474 community dwelling Italians were interviewed. In this analysis, 27003 subjects aged 65 years or older were considered.

**Methods:**

Potentially preventable visits were defined as an emergency department visit that did not result in inpatient admission. Independent variables were classified based on the socio-behavioral model of Andersen-Newman. Descriptive statistics and a logistic regression model were developed.

**Results:**

In the twelve months before the interview 3872 subjects (14.3%) had at least one potentially preventable visit. Factors associated with an increased risk of a potentially preventable visit were older age (75–84 years: OR 1.096, CI 1.001–1.199; 85+years: OR 1.022, CI 1.071–1.391), at least one hospital admission (OR 3.869, IC 3.547–4.221), to waive a visit (OR 1.188, CI 1.017–1.389) or an exam (OR 1.300, CI 1.077–1.570). Factors associated with a lower risk were female gender (OR 0.893, CI 0.819–0.975), area of residence (Center: OR 0.850; CI 0.766–0.943; Islands: OR 0.617, CI 0.539–0.706, South: OR 0.560; CI 0.505–0.622), private paid assistance (OR 0.761, CI 0.602–0.962); a better health-related quality of life (PCS score 46–54: OR 0.744, CI 0.659–0.841; PCS score >55: OR 0.746, CI 0.644–0.865).

**Conclusions:**

Our study identified several characteristics associated with an increased risk of potentially preventable visits to the emergency department. This might allow the development of specific interventions to prevent the access of at risk subjects to the emergency department.

## Introduction

Older patients represent between 12% and 21% of the emergency department (ED) visits and this percentage is expected to increase in the coming decades [1], growing up to about 34% by 2030 [2]. Italian data indicate that older adults already exceed 30% of ED users [3,4]. Older adults have more serious illnesses than other age group on arrival in the ED, as measured by triage acuity, diagnostic work-up, and hospital admission rate, and are also more likely to try to contact their general practitioner or other non-urgent sources of medical care prior to arriving at the ED [5]. Despite this, over 20% of ED attendances are potentially preventable [6], with an increased risk of adverse consequences such as re-admission, hospitalization, mortality [7].

Several studies focused on the potentially preventable ED visit patterns by older adults, but only a few are population-based [5], and many studies are based on administrative data. The hospital administrative data provided a very limited insight other than medical diagnoses, considering that older people attend the ED for a range of reasons that are individual, societal and related to the health services system, as well as strictly clinical [8]. The absence of data on the source population precludes the evaluation of the role of risk factors such as environmental factors or other socio-structural constructs [5].

The aim of our study was to identify socio-demographic characteristics and health care resources use of population-based cohort of community-dwelling older adults admitted to the ED for a potentially preventable visit.

## Materials and methods

Our data come from the Multipurpose Survey “Health conditions and use of health services”, edition 2012–2013. The methods have been previously described in detail [9–12]. In brief, the survey is part of the Italian system of the “Multipurpose Surveys on households” started in 1993 and is repeated every five years. The survey consists of a self-administered paper questionnaire and a face to face interview with paper questionnaire. The sample was selected from the municipal lists of households. using a stratified multi-stage probability design. In the first stage, municipalities were the primary sampling units. The second stage of the sample design involved clustering households from municipality lists. The sampling unit was a household of persons living together, with legal, affective, or family relationships, with regard to the number of persons in the household. Exclusion criteria of the survey were: those household members who had died, residence outside of Italy or in a nursing home, non-existent address, and inability to localize or access the address. Total units selected were 60730 and respondents were 50474. For our analysis, we only considered respondents who were 65 or more years old.

### Variables

Outcome variable was the occurrence of at least one potentially preventable visit to the ED in the twelve months before the interview. This information was available by means of a specific question in the interview: “Did you attend to the emergency department at least one time without an in-hospital admission in the last year?”

To our purpose, we considered the ED visits which did not result in inpatient admission as potentially preventable, according with Agency for Healthcare Research and Quality [13]. This definition has been previously employed in studies regarding ED use [14].

We selected independent variables from the entire survey questionnaire based on the socio-behavioral model of Andersen-Newman that has been extensively used in studies investigating the use of health services, including ED use [5, 15]. The explanatory factors of health care use are classified as predisposing factors, enabling factors, and needing factors [16].

We considered as predisposing factors age, gender, area of residence (North, Central, South and Islands), education (≤ 5 years, 6–8 years; 9–12 years; 13 or more), marital status (single, married, separated/divorced, widow), family composition (formed of a single, a couple with or without children, or a single -mother or father- with children).

Enabling factors included judgment about income (excellent, adequate, low, and insufficient). We also considered the co-payment of public health expenditure. In Italy a private contribute to public health expenditure might be requested depending on age, income and selected conditions such as severe disability or specific diseases (e.g. diabetes, hypertension, cancer). Subjects can be completely exempt, partially exempt or not exempt.

Needing factors included multimorbidity, number of drugs taken every day, disability and the individual health related quality of life (HRQoL). Multimorbidity was defined as the presence of three or more of chronic conditions. It was derived from a predefined list that included diabetes; myocardial infarction; angina pectoris; other diseases of the heart; stroke (ischemic or hemorrhagic); chronic obstructive pulmonary disease, emphysema; cirrhosis; malignant tumor (including lymphoma / leukemia); parkinsonism; dementia, renal failure. Drugs taken every day were considered. Three classes were defined: no drugs, 1–4 medicines every day, 5 or more medicines (polypharmacy).

Disability was assessed using a set of questions based on the International Classification of Impairments Disabilities and Handicaps (ICIDH) of the World Health Organization [17]. Specific disability categories were identified: physical disability (confinement/difficulty walking, lowering oneself, going up/going down, and brushing teeth), personal care (functional autonomy), communication impairment (sight, hearing, and speech), and self-restraint [18].

Health related quality of life was measured using the Short Form 12 with its components (Mental Component Summary, MCS, and Physical Component Summary, PCS), previously used in older adults [19].

In addition, we considered the health care resources use other than the ED. Health care resources included the number of general practitioner visits, specialist visits and in-hospital admissions, and blood or urine tests and instrumental tests (i.e. X-ray, ultrasound, magnetic resonance, CT-scan, ECG). We considered the waive of an exam or a specialist visit for all reasons and for a too long waiting list. The use of a home care services (e.g. nurse and public or private assistants) was recorded. The presence of a private paid assistance was also recorded. A private paid assistance is a man or a woman (not a nurse) who gives help in the activity of daily living basic and instrumental. There isn’t a municipality contribution for this help [20].

### Statistical analysis

The frequency and the percentage or the mean and the standard deviation values were reported for all variables, as appropriate. The admission to the ED was considered a binary variable taking the value of 1 or 0, where 1 is a positive and 0 is a negative answer. For each descriptive variable, we tested if a statistical significant difference exists between the two groups; the χ^2^ test was used. Non-categorical variables were compared using Student’s T-test. or ANOVA, as appropriate.

In addition, a logistic regression model was performed to evaluate which covariates would better explain the factors associated with at least one potentially preventable visit. We created a training sample (24303 observations) and a holdout sample (2700 observations) based on the whole sample. The sample method used was a random extraction without repetition, fixing the dimension for the holdout sample equal to the 10% of the whole sample. The model was developed on the training sample and was then tested on the holdout sample. Moreover, we used the stepwise method to select the significant covariates. The selection process used the Wald χ^2^ test, and the significant level to entry and to stay in the model were fixed at a probability equal to 0.25 and 0.05 respectively. We compared the results through ROC Curves, testing differences between the AUC. Furthermore, the Gini’s Coefficient (Somers’D Index) and the Concordance Index were used to test the predictive capability of the model.

Analyses were performed using SAS version 9.3 (SAS Institute Inc).

## Results

We considered 27003 subjects who were aged 65 years or older. Subjects who had at least one potentially preventable visit to ED in the previous twelve months were 3872 (14.3%). Characteristics of the total sample and of the subgroups with and without at least one potentially preventable visit are shown in Table 1.

**Table 1:** Descriptive analysis of the sample: Socio-behavioural model. Socio-demographic characteristics of the total sample and of the subgroups with and without at least one potentially preventable visit to the ED in the previous year.

Potentially preventable visit				
	Total sample N = 27003	None N = 23131	At least one N = 3872	P
Predisposing factors				
**Gender, F (n, %)**	15336 (56.79)	13133 (56.77)	2203 (56.90)	0.890
**Age (years)** (n, %)				<0.001
65–74	13802 (51.11)	12137 (52.47)	1665 (43.00)	
75–84	9678 (35.84)	8122 (35.11)	1556 (40.19)	
85+	3523 (13.05)	2872 (12.41)	651 (16.81)	
**Education (years)** (n, %)				<0.001
None	3648 (13.51)	3077 (13.30)	571 (14.75)	
1–5	13118 (48.58)	11136 (48.19)	1982 (51.19)	
6–8	5005 (18.53)	4347 (18.79)	658 (16.99)	
9–13	3831 (14.19)	3342 (14.44)	489 (12.63)	
>13	1401 (5.19)	1229 (5.31)	172 (4.44)	
**Marital status** (n, %)				<0.001
Single	1831 (6.78)	1632 (7.05)	199 (5.14)	
Married	15510 (57.44)	13417 (58.00)	2093 (54.05)	
Separated/divorced	1092(4.04)	932 (4.02)	160(4.13)	
Widow/widower	8570 (31.74)	7150 (30.91)	1420 (36.67)	
**Household** (n, %)				<0.001
Single	9872 (36.55)	8166 (35.30)	1506 (38.89)	
Couple with children	3528 (13.06)	3078 (13.30)	450 (11.62)	
Couple without children	12189 (45.13)	10517 (45.46)	1672 (43.18)	
Single parent	1614 (5.97)	1370 (5.92)	244 (6.30)	
**Area of residence** (n, %)				<0.001
North	11867 (43.95)	9919 (42.88)	1948 (50.31)	
Center	5133 (19.011)	4366 (18.87)	767 (19.81)	
South	6911 (25.59)	6138 (26.53)	773 (19.96)	
Islands	3092 (11.45)	2708 (11.70)	384 (9.92)	
**Enabling factors**				
**Self rated Income** (n, %)				<0.001
Excellent	519 (1.92)	467 (2.01)	52 (1.34)	
Adeguate	16196 (59.98)	13989 (60.47)	2207 (57.00)	
Low	9156 (33.91)	7746 (33.48)	1410 (36.42)	
Insufficient	1132 (4.19)	929 (4.01)	203 (5.24)	
**Copayment to health care services** (n, %)				<0.001
Completely exempt	6422 (23.78)	5829 (25.19)	593 (15.31)	
Partially exempt	14119 (52.29)	11781 (50.93)	2338 (60.38)	
not exempt	6462 (23.93)	5521 (23.86)	941 (24.30)	
**Needing factors**				
**At least one chronic condition**	12025 (44.53)	9656 (41.74)	2369 (61.18)	<0.001
**Multimorbidity**	11553 (42.78)	9621 (41.59)	2295(59.19)	<0.001
**Drugs taken everyday (n,%)**				<0.001
0 (n, %)	5032(18.63)	4680 (20.23)	352(9.09)	
1–4 (n, %)	15475 (57.31)	13483 (58.28)	1992 (51.44)	
5+ (n, %)	6496 (24.06)	4968 (21.47)	1528 (39.47)	
**Disability**				
Any type(n, %)	5386 (19.95)	4195 (18.13)	1191 (30.75)	<0.001
Physical (n, %)	2756 (10.21)	2097 (9.06)	659 (17.01)	<0.001
Personal care (n, %)	3524 (13.05)	2676 (11.56)	848 (21.90)	<0.001
Communication impairment (n, %)	1408 (5.21)	1107 (4.78)	301 (7.77)	<0.001
Self restraint (n,%)	2568 (9.51)	1958(8.46)	610 (15.75)	<0.001
**PCS (mean, SD)**	42.6±11.89	51.9±11	37.8±12.1	<0.001
<32 (n, %)	6814(25.23)	5274(22.80)	1540(39.77)	<0.001
32–45 (n, %)	6978(25.84)	5870(25.37)	1108(28.61)	
46–54 (n, %)	7617(28.20)	6842(29.57)	775(20.01)	
≥55 (n, %)	5594(20.71)	5145(22.24)	449(11.59)	
**MCS (mean, SD)**	46.81+10.8	49.8±11.3	43.9±11.8	<0.001
<40 (n, %)	7081(26.22)	5620(24.29)	1461(37.73)	<0.001
40–49 (n, %)	6501(24.07)	5613(24.26)	888 (22.93)	
50-55(n, %)	7160(26.51)	6343(27.42)	817(21.10)	
56+ (n, %)	6261(23.18)	5555(24.01)	706(18.23)	

SD = standard deviation; PCS = Physical Component Summary; MCS = Mental Component Summary

There were relevant differences regarding the area of residence, income, comorbidity, polypharmacy and disability. Furthermore, health-related quality of life was lower in ED users, both in the physical and in the mental components.

The use of health care services is summarized in Table 2. About half of the subjects who had at least one clinical visit (by a specialist or a general practitioner), a diagnostic test or a blood exam attended the ED for a potentially preventable visit, while 30% of subjects who attended in ED were hospitalized at least once in the previous year. A private paid assistance was used by 7% of subjects. Among them, 27% had a potentially preventable visit to the ED. A higher percentage of potentially preventable visits was also recorded among subjects who waived a specialist visit or a diagnostic test. Among these subjects, approximately 20% had been waiting about 15 days, and 4% up to 61 days. Similarly, among those who had to give up a diagnostic test due to the long waiting list, about 24% went to the ED.

**Table 2:** Descriptive analysis of the sample: Health care resources use. Health care resources use of the total sample and of subgroups with and without at least a potentially preventable visit in ED in the previous year.

	Potentially preventable visit			
	Total sample N = 27003	None N = 23131	At least one N = 3872	p
At least one clinical visit in the last 4 weeks (n, %)	13651(50.55)	11140 (48.16)	2511(64.85)	
General practitioner visit in the last 4 weeks (n, %)	10347(38.31)	8471(36.62)	1876(48.45)	<0.001
**In the last 12 months**				
Visits by a specialist (n, %)	17791(65.88)	14552(62.91)	3239(83.65)	<0.001
Blood exams (n, %)	20017(74.10)	16626 (71.87)	3391(87.57)	<0.001
Instrumental tests (n, %)	13418(49.69)	10545 (45.58)	2873(74.19)	<0.001
**Hospital admissions**				
Previous three months(n, %)	1516(5.61)	900 (3.89)	616(15.90)	
Previous twelve months(n, %)	4097(15.17)	2486(10.74)	1161 (29.98)	
**Home care** (n, %)	1915(7.09)	1382(5.97)	533 (13.76)	<0.001
Private paid assistance (n, %)	1186 (4.39)	779(3.36)	257(6.63)	
**To waive a visit** (n, %)	2240(8.29)	1178(5.09)	462(11.93)	<0.001
**waiting list too long** (n, %)	864(3.19)	691(2.98)	173(4.46)	<0.001
**To waive an exam** (n, %)	1395(5.16)	1090(4.71)	305(7.87)	<0.001
**Waiting list too long** (n, %)	567(2.09)	432 (1.86)	135(3.48)	<0.001

The stepwise logistic regression model (Table 3) showed that older age, to be widow or separated, the presence of comorbidity and polypharmacy increase the probability of having at least one potentially preventable visit. The use of health care services (diagnostic test, laboratory blood exams, a hospital admission in the previous year) and waiving for a visit or exam also increase the probability of admission to emergency services. On the other hand, factors associated with a lower probability to have a potentially preventable visit are female gender, living in the South of Italy or in the Islands, having a private paid assistance, and a better health related quality of life.

**Table 3:** Stepwise logistic regression analysis. Stepwise logistic regression analysis of the variables associated with at least one potentially preventable visit in emergency department in the previous 12 months.

Variable	OR	IC 95%
**Gender (female)**	**0.893**	**0.819–0.975**
**Age (years)**		
65–74	1	
75–84	1.096	1.001–1.199
85+	1.022	1.071–1.391
**Area of residence**		
North	1	
Center	0.850	0.766–0.943
South	0.560	0.505–0.622
Islands	0.617	0.539–0.706
**Marital status**		
Single	1	
Married	1.216	1.020–1.449
Separated/divorced	1.293	1.002–1.667
Widow	1.312	1.095–1.573
**Co-payment to health care services**		
Completely exempt	1	
Partially exempt	1.217	1.089–1.359
not exempt	1.065	0.940–1.207
**At least one chronic condition**	1.209	1.102–1.326
**Drugs taken everyday**		
0	1	
1–4	1.185	1.034–1.357
5+	1.445	1.239–1.684
**At least one clinical visit**	1.158	1.063–1.261
**In the last 12 months**		
Visits by a specialist	1.265	1.130–1.417
Blood exams	1.225	1.086–1.382
Diagnostic tests	2.005	1.821–2.207
**Hospital admissions (previous year)**	3.869	3.547–4.221
**Home care**	1.367	1.144–1.633
**Private paid assistance**	**0.761**	**0.602–0.962**
**To waive a visit**	1.188	1.017–1.389
**To waive an exam**	1.300	1.077–1.570
**PCS**		
<32	1	
32–45	0.928	0.836–1.030
46–54	0.744	0.659–0.841
55+	0.746	0.644–0.865

Variables excluded from the stepwise analysis: educational level, MCS score

The ROC Curves built on the training and holdout sample show good AUC values (respectively equal to 0.7659 and 0.7509); the Gini Index (0.532) shows as well the adequacy of the model.

To understand the role of gender in determining the risk to attend ED, we carried out a secondary analysis comparing some characteristics of men and women in the whole sample. Compared with men, women made more often one or more medical visits in the previous four weeks (51.7% vs 48.9%, respectively, p <0.001), also for a clinical health check (14.5% vs 13.5, respectively, p <0.001), and specialist visits (50.6% vs. 48.5%, p = 0.007). Women were more disabled than men (24.4% vs 14.2%, p <0.001), particularly in mobility (12.8% vs. 6.9%, p <0.001), in individual confinement (12.2% vs. 5.9%, p <0.001) and in the activities of daily living (16.3% vs. 8.8%, p <0.001), and had more often multimorbidity (50.4% against 32.8% of men, p <0.001). Moreover, they suffered from different types of chronic conditions: the most prevalent diseases in women were Alzheimer's disease (5.4% versus 2.7% in men, p <0.001) and hypertension (56.1% vs 50.5%, p <0.001), while men are more often affected by diabetes mellitus (18.1% vs 16.8%, p = 0.0062), myocardial infarction (9.5% vs 3.9%, p <0.001); chronic obstructive pulmonary disease (13.7% vs 9.7%, p <0.001).

## Discussion

Our study examined the characteristics of the Italian community-dwelling older adults who experienced a potentially preventable ED visit. Subjects who had at least one visit were older, widow or separated, lived more often in Northern Italy. These subjects were disabled, had a higher number of chronic diseases, and a worse perception of their health status. They used a large number of diagnostic tests and medical visits (GP and specialist), had at least one hospitalization in the previous year. Factors that seem to be associated with a lower probability to attend to the ED were female gender, to live in the South and Islands, to have a private paid assistance and a better perceived of health status.

Only a few studies were carried out on potentially preventable visits in Italian ED.

Barbadoro et al [21] examined non-urgent visits in a sample of young and old patients admitted to a single hospital. They found that a large part of patients suffered from chronic diseases and took medicines everyday. The authors affirm that the Italian primary care system is possibly suffering from the increasing age and comorbidity of citizens, and the increasing perception of diagnostic technologies as the sole offering an appropriate diagnosis, despite the pivotal role given to GP. Bianco et al [22] found that the odds of requiring non-urgent care were significantly higher in patients who present to the emergency department without medical referral and in patients who present with problems of longer duration. Fusco and coll. [3] emphasized the need to develop a new organizational model for delivering primary care to older patients because the ED mission is to treat urgent conditions. These Italian findings are consistent with our data, which show that those who make at least one potentially preventable admission in ED are frequent users of other health care services. A private paid assistance is a key factor in the management of older adults. Italian studies showed that about 20% of older adults, who are severely disabled, i.e. suffer from disability in basic activities of daily living, received assistance from private paid assistants [23]. Our findings are consistent with other authors, who found that having assistance at home is important to prevent hospital and ED admissions [24]. Furthermore, the lack of availability of health services is a main risk factor for the use of the ED, particularly for conditions that could be managed outside the hospital. The use of the ED is considered an indicator of a potential malfunction of the network of primary services. A study by Rust et al on 30.677 adults admitted to the ED showed that one person in three that went to the ED, had found obstacles in planning specialist visit or diagnostic test [25]. In surveys, as many as half of patients presenting at the ED for non-urgent reasons cited an inability to get a timely appointment with their healthcare provider as a reason for their visit [26, 27]. In our sample subjects who had failed to see a specialist or to do a diagnostic assessment had an increased probability to access to ED.

In agreement with international data, other risk factors for potentially preventable ED admissions in older adults are older age [28], comorbidity and polypharmacy [29]; area of residence, in reason of the different health care organization, which includes the rate general practitioner/patients [30], and the economic resources [31]. In this respect, it should be noted that the exams and specialist consultation are free in the ED or require only a small contribution if the ED visits are deemed to be non urgent ones. On the contrary, these subjects might be requested to pay diagnostic exams and visits provided in a non urgent setting.

Many studies have also identified the perceived health status as an important factor associated with the consumption of health care resources [32]. Stevens et al found that ED users for non-clinical problems reported a low (21%) or poor perceived health status (19%) [33].

The role of female gender could have different explanations. Despite women are more disabled, they do more prevention than men as shown also in our previous study [34].Furthermore, men suffer more often from diseases considered ambulatory care sensitive conditions, such as diabetes mellitus and chronic obstructive pulmonary disease [13].

### Strengths and limitations

The main strength of our study is the evaluation of a representative sample of the Italian community-dwelling older population, based on a stratified multi-stage probability design. In addition, our data provide a description of socio-demographic characteristics of Italian older ED users. On the other hand, some limitations deserve comments: 1) not all ED visits that result in a discharge are preventable. 2) Other data, i.e. the reason why a subject referred to ED, as well as the gravity or the temporal relationship between the use ED and that of other healthcare services are unknown. 3) Emergency Department admissions and health care resources consumption are self-reported. 4) Finally, the survey collects information at different times. A potentially preventable visit could happen at any time last year, whereas some data, e.g. the variable measuring quality of life, reveal the situation of the patients at the time of the interview (and certainly they were measured after the outcome). In a cross-sectional study the exposure measure can anticipate or come after the outcome. Furthermore, it can’t be excluded that some variables, such as the self-perceived health status and pharmacotherapy, changed after ED admission.

## Conclusions

Our results identified several factors associated with an increased risk to experience a potentially preventable visit in emergency department. Age, area of residence, marital status and chronic conditions are non-modifiable variables that identify specific subgroups of the population who are at high risk of potentially preventable admission to the ED. On the other hand, waiting list and home assistance are modifiable factors that can be targeted by public health intervention.

## References

[1] Braes T, Moons P, Lipkens P, Sterckx W, Sabbe M, Flamaing J, et al Screening for risk of unplanned readmission in older patients admitted to hospital: predictive accuracy of three instruments. Aging Clin Exp Res. 2010; 22:345–51. 21116125 10.1007/BF03324938

[2] Carpenter CR, Heard K, Wilber S, Ginde AA, Stiffler K, Gerson LW, et al Society for Academic Emergency Medicine (SAEM) Geriatric Task Force. Research priorities for high-quality geriatric emergency care: medication management, screening, and prevention and functional assessment. Acad Emerg Med. 2011;18:644–54. doi: 10.1111/j.1553-2712.2011.01092.x 21676064 10.1111/j.1553-2712.2011.01092.x PMC3117251

[3] Fusco M, Buja A, Furlan P, Casale P, Marcolongo A, Baldovin T, et al Older adults in Emergency Department: management by clinical severity at triage. Ann Ig. 2014 26:409–17. doi: 10.7416/ai.2014.2000 25405371 10.7416/ai.2014.2000

[4] Gasperini B, Cherubini A, Fazi A, Maracchini G, Prospero E. Older adults in Emergency Departments: the challenge of undertriage. Intern Emerg Med. 2016;11(8):1145–1147. doi: 10.1007/s11739-016-1503-x 27384767 10.1007/s11739-016-1503-x

[5] Grunier A., Silver MJ., Rochon PA. Emergency Department Use by Older Adults: A Literature Review on Trends, Appropriateness, and Consequences of Unmet Health Care Needs. Medical Care Research and Review. 2011 68:131–155 doi: 10.1177/1077558710379422 20829235 10.1177/1077558710379422

[6] McCusker J, Verdon J.Do geriatric interventions reduce emergency department visits? A systematic review.J Gerontol A Biol Sci Med Sci. 2006;61:53–62. 16456194 10.1093/gerona/61.1.53

[7] McCusker J, Dendukuri N, Tousignant P, et al Rapid two-stage emergency department intervention for seniors: impact on continuity of care. Acad Emerg Med 2003;10(3):233–43. 12615589 10.1111/j.1553-2712.2003.tb01997.x

[8] Faulkner D, Law J. The 'unnecessary' use of emergency departments by older people: findings from hospital data, hospital staff and older people. Aust Health Rev. 2015;39(5):544–51. doi: 10.1071/AH14185 25913422 10.1071/AH14185

[9] ISTAT, Information system on quality of statistical production processes, Approfondimenti. http://siqual.istat.it/SIQual/visualizza.do?id=0071201 Accessed 1st Jan 2017.

[10] ISTAT (Italian National Institute of Statistics). [Health conditions and the use of healthcare services–Year 2012]. Available at: http://www.istat.it

[11] http://www.istat.it/it/files/2014/07/Nota-metodologica-Salute_2013.pdf?title=Tutela+della+salute+e+accesso+alle+cure+-+10%2Flug%2F2014+-+Nota+metodologica.pdf.

[12] Barbadoro P, Cotichelli G, Chiatti C, Simonetti ML, Marigliano A, Di Stanislao F, et al Socio-economic determinants and self-reported depressive symptoms during postpartum period. Women Health. 2012;52:352–68. doi: 10.1080/03630242.2012.674090 22591232 10.1080/03630242.2012.674090

[13] Fingar KR, Barrett ML, Elixhauser A, Stocks C, Steiner CA. Trends in Potentially Preventable Inpatient Hospital Admissions and Emergency Department Visits: Statistical Brief #195. 2015 Nov. 26741014

[14] Lee MJS, Moore L, Perry J, Daoust R, Griffith L, Worster A, et al Return to the ED and hospitalisation following minor injuries among older persons treated in the emergency department: predictors among independent seniors within 6 months Age Ageing 2015 44 (4): 624–629 doi: 10.1093/ageing/afv054 25944869 10.1093/ageing/afv054 PMC4476849

[15] Kaskie B, Obrizan M, Jones MP, Bentler S, Weigel P, Hockenberry J. et al Older adults who persistently present to the emergency department with severe, non-severe, and indeterminate episode patterns. BMC Geriatrics 2011 11:65 doi: 10.1186/1471-2318-11-65 22018160 10.1186/1471-2318-11-65 PMC3215637

[16] Andersen RM, Newman JF. Societal and individual determinants of medical care utilization in the United States. Milbank Mem Fund Q Health Soc. 1973;51:95–124. 4198894

[17] International Classification of Functioning, Disability and Health (ICF). World Health Organization, http://www.who.int/classifications/icf/en/ Accessed 1st Jan 2017

[18] ISTAT (2009) La disabilità in Italia Il quadro della statistica ufficiale 978-88-458-1628-4, Argomenti n. 37

[19] Jakobsson U. (2007) Using the 12-item Short Form health survey (SF-12) to measure quality of life among older people. Aging Clin Exp Res. 2007 12;19:457–64. 18172367 10.1007/BF03324731

[20] Cherubini A. Gasperini B., Orso F., Di Bari M. Italy. In: Palmore E.B. Whittington F., Kunkel S, editors. International Handbook on Aging. Current Research and Developments.Santa Barbara, California: ABC-CLIO, LLC; 2009 Pp 309–320.

[21] Barbadoro P, Di Tondo E, Menditto VG, Pennacchietti L, Regnicoli F, Di Stanislao F, D'Errico MM, Prospero E. Emergency Department Non-Urgent Visits and Hospital Readmissions Are Associated with Different Socio-Economic Variables in Italy. PLoS One. 2015 15;10(6):e0127823 doi: 10.1371/journal.pone.0127823 26076346 10.1371/journal.pone.0127823 PMC4468197

[22] Bianco A, Pileggi C, Angelillo IF. Non-urgent visits to a hospital emergency department in Italy. Public Health. 2003;117(4):250–5. doi: 10.1016/S0033-3506(03)00069-6 12966745 10.1016/S0033-3506(03)00069-6

[23] Di Bari M, Pecchioli A, Mazzaglia G, Marini M, Maciocco G, Ferrucci L, Marchionni N. Care available to severely disabled older persons living at home in Florence, Italy. Aging Clin Exp Res. 2008 2;20(1):31–9. 18283226 10.1007/bf03324745 PMC2646101

[24] Hominick K, McLeod V, Rockwood K. Characteristics of Older Adults Admitted to Hospital versus Those Discharged Home, in Emergency Department Patients Referred to Internal Medicine. Can Geriatr J. 2016 31;19(1):9–14. doi: 10.5770/cgj.19.195 27076860 10.5770/cgj.19.195 PMC4815936

[25] Rust G, Ye J, Baltrus P, Daniels E, Adesunloye B, Fryer GE. Practical barriers to timely primary care access: impact on adult use of emergency department services. Arch Intern Med. 2008 11;168(15):1705–10. doi: 10.1001/archinte.168.15.1705 18695087 10.1001/archinte.168.15.1705

[26] Afilalo J, Marinovich A, Afilalo M, Colacone A, Léger R, Unger B, Giguère C. Nonurgent emergency department patient characteristics and barriers to primary care. Acad Emerg Med. 2004;11(12):1302–10. Erratum in: Acad Emerg Med. 2005 Jan;12(1):12. doi: 10.1197/j.aem.2004.08.032 15576521 10.1197/j.aem.2004.08.032

[27] Atenstaedt R, Gregory J, Price-Jones C, Newman J, Roberts L, Turner J. Why do patients with nonurgent conditions present to the Emergency Department despite the availability of alternative services? Eur J Emerg Med. 2015;22(5):370–3. doi: 10.1097/MEJ.0000000000000224 25405462 10.1097/MEJ.0000000000000224

[28] Shah MN, Glushak C, Karrison TG, Mulliken R, Walter J, Friedmann PD, Hayley DC, Chin MH. Predictors of emergency medical services utilization by elders. Acad Emerg Med. 2003 10(1):52–8. 12511315 10.1111/j.1553-2712.2003.tb01976.x

[29] Cherubini A, Eusebi P, Dell'Aquila G, Landi F, Gasperini B, Bacuccoli R, Menculini G, Bernabei R, Lattanzio F, Ruggiero C. Predictors of hospitalization in Italian nursing home residents: the U.L.I.S.S.E. project. J Am Med Dir Assoc. 2012;13(1):84.e5–10. 10.1016/j.jamda.2011.04.001 21621481

[30] Shah MN, Bazarian JJ, Lerner EB, Fairbanks RJ, Barker WH, Auinger P, Friedman B. The epidemiology of emergency medical services use by older adults: an analysis of the National Hospital Ambulatory Medical Care Survey. Acad Emerg Med.2007;14(5):441–7. doi: 10.1197/j.aem.2007.01.019 17456555 10.1197/j.aem.2007.01.019

[31] Searing LM, Cantlin KA. Nonurgent Emergency Department Visits by Insured and Uninsured Adults. Public Health Nurs. 2016; 33(2):93–8. doi: 10.1111/phn.12238 26464116 10.1111/phn.12238

[32] Aminzadeh F, Dalziel WB. Older adults in the emergency department: a systematic review of patterns of use, adverse outcomes, and effectiveness of interventions. Ann Emerg Med. 2002; 39(3):238–47. 11867975 10.1067/mem.2002.121523

[33] Stevens TB, Richmond NL, Pereira GF, Shenvi CL, Platts-Mills TF. Prevalence of nonmedical problems among older adults presenting to the emergency department. Acad Emerg Med. 2014; 21(6):651–8. doi: 10.1111/acem.12395 25039549 10.1111/acem.12395

[34] ISTAT Rapporto annuale Istat 2012. La situazione del Paese. 2012. http://www.istat.it/storage/Rapporto_annuale_2012/epub/Rapporto%20Annuale%202012%20-%20ISTAT.epub Accessed 1st Jan 2017

